# Deformation and Fracture Failure of a High-Speed Long Rod Intercepted by Linear Explosively Formed Penetrators Sequence

**DOI:** 10.3390/ma13225086

**Published:** 2020-11-11

**Authors:** Yishu Li, Zhonghua Huang, Anshun Shi, Xiangqun Xu, Sanmin Shen, Han Liu

**Affiliations:** 1 School of Mechatronical Engineering, Beijing Institute of Technology, Beijing 100081, China; liuh@bit.edu.cn; 2 No.52 Institute of China Ordnance Industries, Yantai 264003, China; shianshun111@126.com (A.S.); xxq9420@163.com (X.X.); 3 Key Laboratory of Instrumentation Science and Dynamic Measurement, Ministry of Education, North University of China, Taiyuan 030051, China; shensanming@nuc.edu.cn

**Keywords:** high-speed long rod, linear shaped charge, linear explosively formed penetrator, fracture, yaw

## Abstract

The fracture failure of a high-speed long rod has historically been a challenge. Since the flying plate and flying rod have a relatively low velocity, it is challenging to achieve a multi-stage fracture of the high-speed long rod within the range of existing technology. In this paper, the linear explosively formed penetrators (LEFPs) sequence with a stable flight velocity of 850 m/s were used to cut a high-speed long rod. We investigated the deformation and fracture of Φ10 mm tungsten alloy long rods having different length-diameter ratios (20, 26, 35) and different speeds (1200, 1400, 1600 m/s) by employing the LEFPs sequence with different spacings (0–40 mm) and different interception angles (30°, 60°). In the meantime, the fractured rods movement pattern was recorded with a high-speed camera to elucidate the change law of the length, speed, linear momentum, and angular momentum of fractured rods. It was found that the length loss rate of the fractured rods is as high as 27%. The fractured rods rotated around the center of mass, and the vertical speed change could reach up to 18% of the muzzle velocity of the long rod, and the greatest reduction of horizontal speed and momentum could reach 37%. The longer the interaction time between LEFPs sequence and the long rod, the more beneficial the failure of the long rod. The application of LEFPs sequence solved the difficult problem of disabling the high-speed long rod, and the quantitative analysis of the fracture failure of the long rod had an important sense for studying the terminal penetration effect of the fractured rods.

## 1. Introduction

The fracture failure of a high-speed long rod has always been a challenge. In the range of existent technology, the dynamic flying plates or flying rods were concerned with applying the interception of a high-speed long rod, resulting in deformation or fracture of a high-speed long rod. E. Lidén et al. studied the shape of the fractured rod after the interaction of a 60° inclined steel flying plate and a long rod [1]. Its influencing factors include the length-to-diameter ratio (15, 30, 45), the thickness of the steel plate (0.5, 1, 1.5 times of the projectile diameter), long-rod projectile velocity (1500, 2000, 2500 m/s), and steel plate velocity (−300 to 300 m/s) [2,3]. Afterwards, the process from the deformation to the fracture of the long rod was reproduced by numerical simulation and the fracture mechanism of the long rod was analyzed [4]. After defining the dimensionless parameters of interaction between the long rod and flying plate (the ratio of the vertical movement velocity of the fractured rod to the horizontal sliding velocity), E. Lidén et al. concluded that the greater the value of this parameter, the larger the number of fractured segments of the long rod [5]. Shin H. et al. also used the numerical simulation method for investigating the effects of plate velocity and long rod impact velocity on residual kinetic energy and exploring the interaction mechanism of the long rod with a flying plate [6]. In addition to the effect of the flying plate on the long rod, E. Lidén et al. also carried out simulation and experimental research on the influence of the flying rod velocities and hitting points on the long-rod projectile [7]. Yo-Han Yoo et al. also used numerical simulation methods to study the effect of the diameter of the flying rod, the distance and position of the collision on the failure of the long rod, as well as the effect on the residual penetration depth after the deformation of the long rod [8,9]. Inspired by the flying rod intercepting the high-speed long rod, Joo J. et al. replaced the flying rod with the linear explosively formed penetrator (LEFP) and investigated the collision effect against a long rod by a LEFP through a series of numerical analyses [10]. As the detonation product of a linear-shaped charge (LSC), the stable flight velocity of LEFP is significantly higher than that of the flying plate or flying rod. Lim. et al. established the steady state analytical equation of motion of LSC jet after LSC detonation based on the Birkhoff theory and studied the shape and velocity of LSC detonation products [11,12,13,14,15,16]. In the literature [17,18,19,20,21], the effects of different structural parameters and material properties of LSC on its penetration properties were studied. After designing the LSC with an arc-shaped liner, Ruijun Gou et al. established a theoretical model of the LEFP detonation at different initiation positions and discussed the factors affecting its penetration depth [21,22]. The process of LEFP truncating the long rod can be regarded as the thermomechanical loading process, and the occurrence and propagation of cracks lead to the failure of the long rod. A. Singh et al. studied the effect of thermomechanical loading on an edge crack of finite length in an infinite orthotropic strip and obtained the analytical expressions for the stress intensity factor (SIF) at the crack tip [23]. In the literature [24,25], numerical simulations were performed to establish the role of the residual stresses generated after fatigue precaching, considering different Kmax-levels. The implications of the finite element results for fracture criteria based on critical stress or strain, or both, was discussed with respect to the transition of fracture mode and the angle of initial crack-growth [26].

To address the challenge of multi-segment fracture failure of the high-speed long rod, the LEFPs sequence was adopted to intercept the high-speed long rod in this article. First, we obtained the forming shape and flight speed of LEFP through the LSC detonation test. Afterwards, the influence of LEFP interception angles (30°, 60°), spacing (0, 10, 20, 30, and 40 mm), long rod length-to-diameter ratios (20, 26, 35), and velocity (1200, 1400, 1600 m/s) on the interaction between tungsten long-rod (Φ10 mm) and LEFP were studied experimentally. Finally, this article put up a quantitative analysis on the otherness of before and after fracture failure about the length loss rate, velocity change, momentum change, angular momentum change, and constructed concern between the influence factors and the fracture failure of the long rod. This work has an important significance for the fracture failure study of a high-speed long rod.

## 2. Experiment

**Figure 1:**
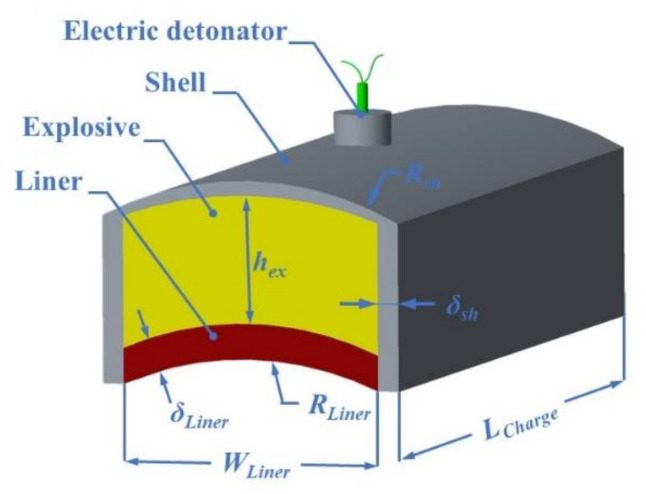
Schematic diagram of a linear-shaped charge (LSC).

LSC is a strip-shaped charge structure, which is composed of a metal liner, insensitive explosive, and non-metallic shell. The LSC detonated at the center point. The center of the shell is perforated to insert the detonator to ensure that the detonator is in good contact with the explosive, as shown in Figure 1. *R*_Liner_, δ_Liner_, *R*_sh_, *δ*_sh_, *W*_Liner_, *h*_ex_, and *L*_C__hanrge_ are the liner inner diameter, the liner thickness, the outer diameter of the shell base, shell thickness, charge width, charge height, and charge length, respectively.

### 2.1. Linear-Shaped Charge (LSC) Test Preparation

**Figure 2:**
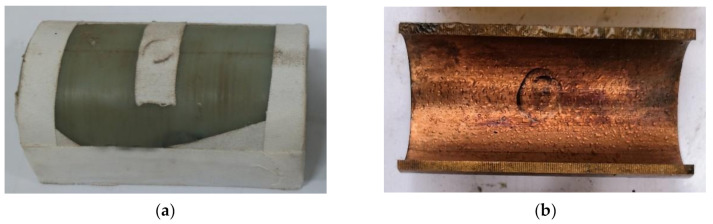
The linear-shaped charge used in the experiment: (**a**) Main charge; (**b**) arc liner used in the experiment.

Figure 2 shows the LSC used in the experiment. Figure 2a is the main charge of LSC, and Figure 2b is the copper liner of LSC. Table 1 shows the related parameters of the LSC. The length of the LSC is 100 mm, in which 96 g of insensitive explosives are loaded between the liner and the shell. The detonation velocity of explosive is 5880 m/s, with a charge density of 1.1 g/cm^3^. The material parameters of the liner are shown in Table 1.

### 2.2. X-ray Test Setup of Linear-Shaped Charge

**Figure 3:**
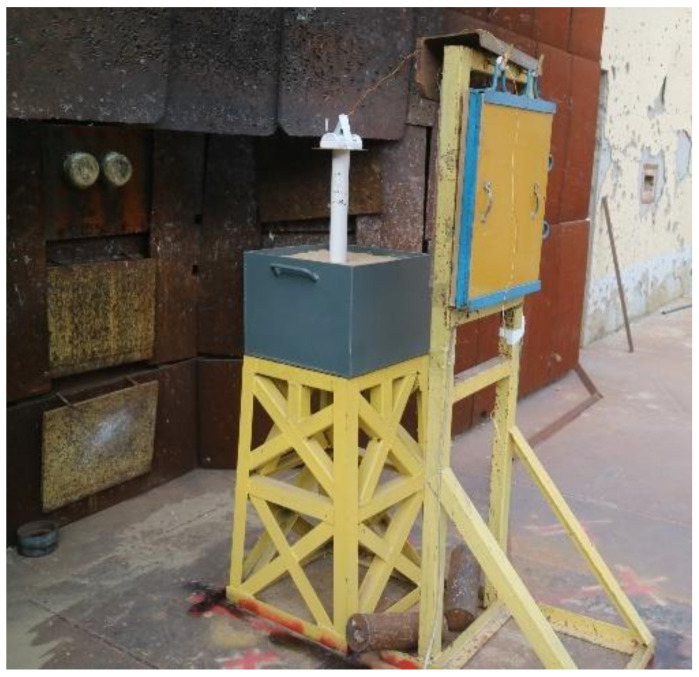
Flash X-ray experimental setup diagram.

To obtain the shape and velocity of the LEFP after the LSC detonation, an X-ray test was carried out. Figure 3 is a layout diagram of a LSC X-ray test. The LSC was placed on a PVC cylinder that is situated in a sandbox. The height of the PVC cylinder was 300 mm. The LSC, PVC cylinder, and sandbox were placed between the X-ray exit and the imaging device. The imaging device was composed of an imaging plate and a protective cover.

### 2.3. Interception Experiment of Dynamic Long Rod with Multiple Linear-Shaped Charges

**Figure 4:**
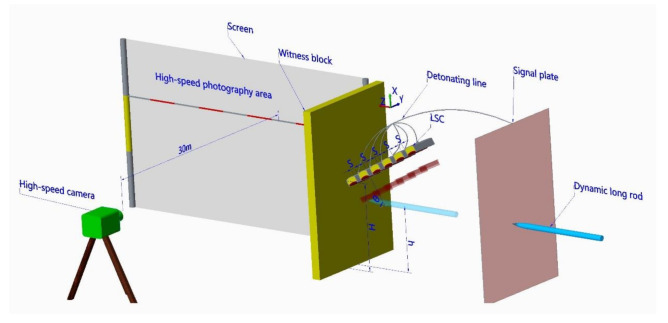
Schematic diagram of the dynamic test layout.

Multiple LSCs were arranged side by side at a certain inclination angle θ above the striking direction of the long-rod projectile, in which the spacing between adjacent LSCs was *s*, and the lowest linear-shaped charge was 200 mm above the ground (*H* = 200 mm). The signal plate was placed in front of the LSC. The backplane was a SHY685NS steel plate with a thickness of 30 mm, which is placed behind the LSC. The distance between the detonating plate and the backplane was 390 mm. The body of the long rod projectile was a tungsten alloy with a diameter of 10 mm. The striking height of the long-rod projectile was *h*. The long rod first hit the signal board, which transmits electrical signals to the electric detonators. The electric detonators simultaneously detonated multiple LSCs, and then formed the LEFPs sequence, resulting in the fracture of the long rod. The fractured rods penetrated the witness plate, which not only recorded the direction of penetration of the fractured rods, but also effectively isolated the scattering of detonation products and part of the detonation glare, facilitating the photographing of motion morphology of the fractured rods with a high-speed camera. The high-speed camera was placed 30 m away from the curtain. The test layout is shown in Figure 4.

The parameters describing the damage and fracture performance of long rod include the change in mass of the fractured rod (∆*m*/*m*_proj_), the change in length (−∆*L*/*L*), the number of fractures (*n*), and the ratio of the longest length of the fractured rod (*L*_max_/*L*). To better describe the shape of the long rod fracture and damage after being intercepted, the velocity, momentum, and angular momentum of the fractured rods need to be analyzed. The speed change of the fractured rods can be described as:

(1) $$\left|\Delta V_{X}\right|=\overset{n}{\underset{Xi=n}{\sum }}\left|V_{\mathrm{Xi}}\right|=\overset{n}{\underset{Xi=n}{\sum }}\left|\frac{r_{{\mathrm{Xi}}_{2}}-r_{{\mathrm{Xi}}_{1}}}{t_{2}-t_{1}}\right|$$

(2) $$\Delta V_{Z}=\overset{n}{\underset{Zi=n}{\sum }}\Delta V_{\mathrm{Zi}}=\overset{n}{\underset{Zi=n}{\sum }}\left(V_{\mathrm{Zi}}-V_{\mathrm{proj}}\right)$$

(3) $$V_{\mathrm{Zi}}=\frac{r_{{\mathrm{Zi}}_{2}}-r_{{\mathrm{Zi}}_{1}}}{t_{2}-t_{1}}$$

where ($r_{{\mathrm{Xi}}_{1}},r_{{\mathrm{Yi}}_{1}},r_{{\mathrm{Zi}}_{1}})$ and ($r_{{\mathrm{Xi}}_{2}},r_{{\mathrm{Yi}}_{2}},r_{{\mathrm{Zi}}_{2}})$ represent the three-dimensional coordinates of fractured rod *i* at times *t*_1_ and *t*_2_, respectively. The number of fractured rods is *n*.

(4) $$\Delta V=\underset{i}{\sum }\left(m_{i}\Delta V_{i}\right)/m_{\mathrm{rps}}$$

where *m*_i_, Δ*V*_i_, and $m_{\mathrm{rps}}=\underset{i}{\sum }m_{i}$ represent the mass of the fractured rod *i*, the velocity change value of the fractured rod center of mass, and the total mass of the fractured rods, respectively. The momentum change of the fractured rod is described as:

(5) $$\Delta P=\underset{i}{\sum }m_{i}\left(V_{\mathrm{proj}}+\Delta V_{i}\right)-P_{\mathrm{proj}\;}\;$$

where *P*_proj_ = *m*_proj_ × *V*_proj_ is the momentum of the long rod. *m*_proj_ and *V*_proj_ represent the mass and velocity of the long rod, respectively. The angular momentum of the fractured rods is described as:

(6) $$\Delta H=\underset{i}{\sum }[J_{i}\Delta \omega_{i}+\left(r_{i}-r_{\mathrm{rps}}\right)\times m_{i}\Delta V_{i}\;$$

where *J*_i_ and Δ*ω*_i_ are the moment of inertia and angular velocity of the fractured rod *i*, respectively. *r*_i_ is the moving distance of the center of mass of the fractured rod *i*, whereas *r*_rps_ is the moving distance of the center of mass of all fractured rods.

## 3. Results

### 3.1. X-ray Test Results

**Figure 5:**
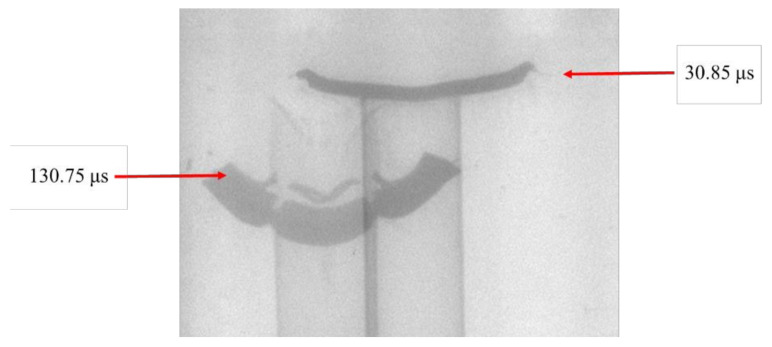
Flash X-ray experiment images at a typical time.

**Figure 6:**
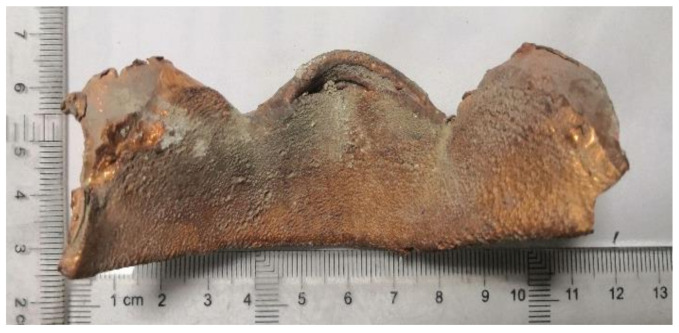
Linear explosively formed penetrator (LEFP) shape.

Figure 5 shows X-ray images captured at 30 and 130 μs after detonating the LSC. It can be seen that the LSC liner began to flip at 30 μs, and the detonation product has completed flipping at 130 μs to reach a stable flight status. The flight velocity of the LSC detonation product was calculated by X-ray photography to be about 850 m/s. Figure 6 shows the LSC detonation product collected in the X-ray experiment sandbox. It can be seen that the shape of the overturned molding of the liner was basically consistent with the X-ray images.

### 3.2. Test Results of LEFP Interception High-Speed Long Rod

When the linear-shaped charge (LSC) detonated, the resulting LEFP intercepted the dynamic long rod. Through high-speed photography, photos of the dynamic long rods deformation and fracture were captured. The test results were obtained by analyzing the photos. In the test, the length-to-diameter ratios of the dynamic long rods were 20, 26, and 35, and the velocity were 1200, 1400, and 1600 m/s, respectively. The detonation delay of the signal board was 10 μs. The inclinations of the linear-shaped charge in the test θ were selected as 30 and 60°. The spacing of the LSCs was also selected as 0, 10, 20, 30, and 40 mm. The test results are shown in Table 2. The ratios of multi-section fractured rods to the long rod and the variations in velocity, linear momentum, and angular momentum of the multi-section fractured rods are calculated and listed in Table 3.

## 4. Discussion

### 4.1. The Fractured Rods and Bullet Hole Shape

**Figure 7:**
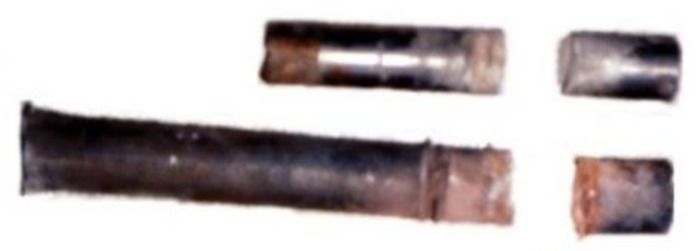
Fractured rods diagram.

After the LEFPs sequence hit the long rod, the long rod fractured. Figure 7 shows a photograph of the tungsten alloy long rod fractured by the LEFP interception. The penetration trace by copper LEFP remained at the cut of the tungsten alloy fractured rod, in which the cut surface of the fracture is relatively flat.

**Figure 8:**
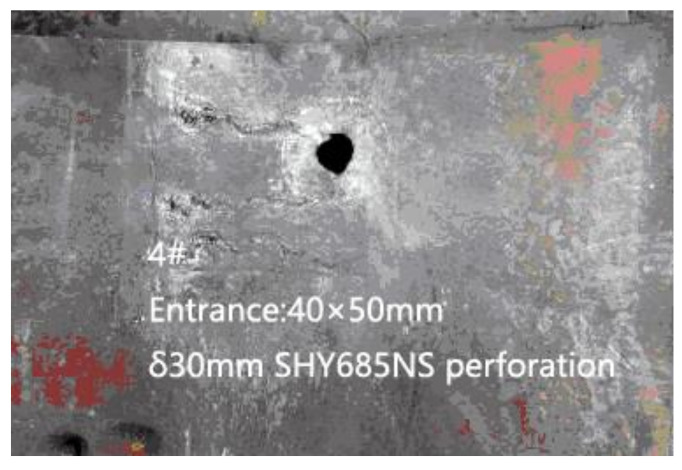
Damage results of witness block (4#).

**Figure 9:**
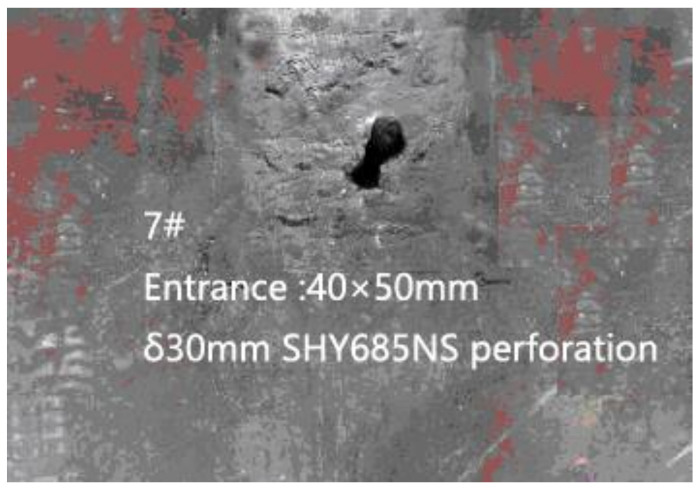
Damage results of witness block (7#).

When the fractured rods passed through the backplane, the bullet hole appeared as a single hole expansion or multiple holes. Figure 8 is the photograph of the fractured bar penetrating the backplane in the fourth test, in which the bullet hole size was 40 mm × 50 mm. Figure 9 is the photography screenshot of the fractured bar penetrating the backplane in the seventh test, which has a bullet hole size of 40 mm × 70 mm. It can be seen that the size of the bullet holes on the backplane was all larger than the diameter of the dynamic long rod by 10 mm. Under the action of multiple LEFPs, the dynamic long rod all fractured, and the moving direction of the fractured rods changed to a certain extent, deviating from the initial trajectory direction and enlarging the incident hole of the backplane.

**Figure 10:**
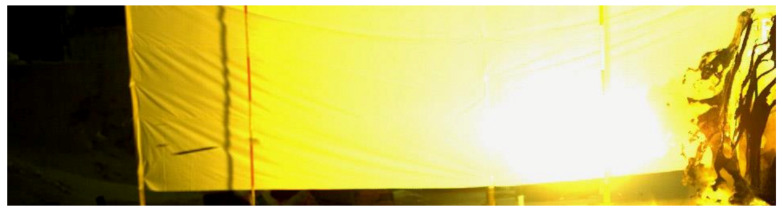
High-speed photography screenshots of the fourth dynamic test (4#).

**Figure 11:**
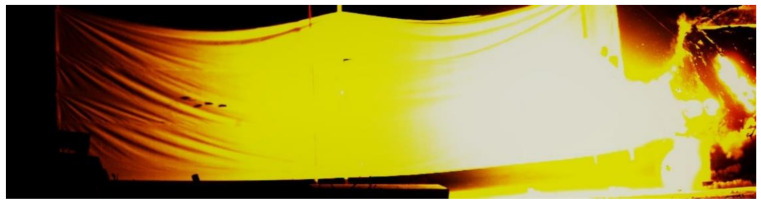
High-speed photography screenshots of the seventh dynamic test (7#).

Figure 10 shows a high-speed photography screenshot of the fractured rod at 24.1 ms in the fourth test. It can be seen that the head of the long rod was fractured, and the moving direction of the two fractured rods shifted to a certain extent. Figure 11 is a high-speed photography screenshot of the fractured rod at 19.9 ms in the 7th test. It can be seen that the long rod fractured into four relatively even fractures. The four fractured rods were scattered around with different moving directions. The long rod fractured into a large number of fractures with various moving directions, resulting in multiple craters on the backplane by the fractured rod, which increases the area of the bullet holes on the backplane.

Therefore, it can be concluded that the number, length, and deflection angle of the fractured rod affect the opening shape of the backplane to a certain extent, which in turn affects the penetration performance of the fractured rod. In the following section, the influencing factors such as the length-to-diameter ratio, velocity of the long rod, and LSC spacing on the fractured rod shape are analyzed and studied.

### 4.2. Effect of the Length-Diameter Ratio of the Long Rod

**Figure 12:**
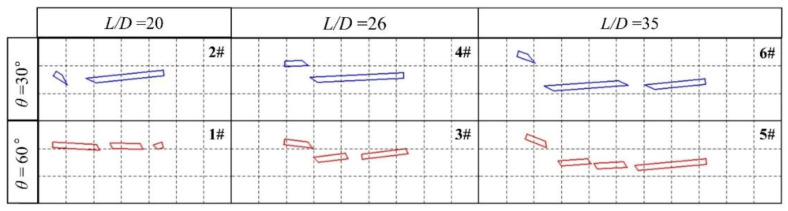
Location pattern of the residual fractured rods after the interaction of long rods having different *L*/*D* ratios with different interception angles of LEFP (26 ms).

Figure 12 shows a screenshot of the position of the fractured residual rod on the screen captured by a high-speed camera at 26 ms after the dynamic long rod triggers the signal board, in which each dotted grid represents 50 mm × 50 mm. The velocity of the long rod was 1200 m/s. The length-to-diameter ratios of the long rod from left to right in the photo were *L*/*D* = 20, 26, and 35, and the interception angles from top to bottom were θ = 30° and 60°. The test number was marked in the upper right corner.

**Figure 13:**
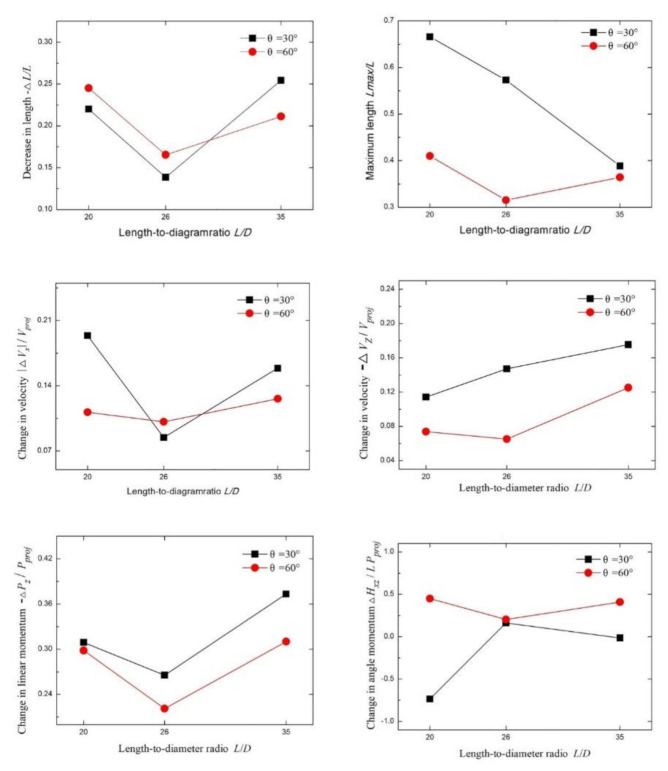
Influence of length to diameter ratio *L*/*D* of the long rod at different inclination angles.

It can be seen from Figure 13 that as the length-to-diameter ratio (L/D) of the long rod increased, the number of fractured rods increased. The main reason is that under the same long rod diameter *D*, with increasing *L*/*D*, the long rod length *L* increased, and the number of LEFPs that can act on the long rod also increased. As a result, the number of fractured rods increased. At the same time, the number of fractured rods at θ = 60° was greater than that at θ = 30°, primarily because the distance *d* between LEFPs and the long rod at θ = 60° is smaller than that at the time of θ = 30°, which causes more LEFPs to contact with the long rod within a certain time and generate more truncated rods.

Since the deflection direction and angle of the fractured rods are affected by the center of mass and the interacting position of the LEFP, the deflection direction of the fractured rod in different positions varied accordingly. The head fractured rod rotates counterclockwise around the center of mass. The tail fractured rod, which is exactly opposite of the head fractured rod, rotates clockwise around the center of mass. The middle-fractured rods are subjected to the LEFP shearing force at the front and rear end position, which rotate clockwise and counterclockwise successively and smoothly around the center of mass with deflection angles smaller than those of head and tail fractured rods.

Figure 13 shows the trend of the evaluation of the fractured rods. The data in the figure are based on the data of entries 1–6 in Table 3. After the long rod fractured, the length of the rod changed to a certain extent. −Δ*L*/*L* is the length loss rate after the long rod collides the LEFP. The number of fractured rods determines the change in the total length of the fractured rods (−∆*L*). In other words, the larger the number of fractured rods, the greater the value of −∆*L*. When *L*/*D* was 20 and 26, the number of fractured rods was identical, with increasing *L*_proj_, the value of −∆*L*/*L* decreased. When *L/D* was 35 and the number of fractured rods increased, with rising −∆*L*, the value of −∆*L*/*L* was greater than the value of *L*/*D* = 26.

*L*_max_ is the length of the longest fractured rod. When θ = 60°, the value of *L*_max_/*L* was less than θ = 30°; meanwhile, when θ = 30°, *L*_max_/*L* showed a downward trend with the increase of *L*/*D* primarily since the number of fractured rods increases and the length of the longest fractured rod also decreases. At the same time, the *L*_max_/*L* value had certain randomness, which depends on the intersection of the long rod and LEFP to a certain extent. Since the penetration depth of the longest fractured rod is related to its length, the smaller the *L*_max_/*L* value, the lower the subsequent penetration performance of the fractured rod.

*V*_x_ is the velocity component in the x-axis direction when the fractured rod is deflected by the LEFP. *V*_x_ depends on the length of the fractured rod and the position of the fractured rod impacted by LEFP. The longer the length of the fractured rod, the larger the inertia and the smaller the *V*_x_. The rotation angle of the fractured rod depends on the position of fractured rod impacted by LEFP, implying that the positive and negative values of *V*_x_ are different. |Δ*V*_x_| is the absolute value of the velocity change of all fractured rods. The larger the |Δ*V*_x_| value, the greater the longitudinal velocity obtained by all fractured rods. The instability of fractured rods is positively correlated with the angle of the fractured rods from the original penetration direction. It can be seen that when *L*/*D* was 20 and 35, |∆*V*_x_|/*V*_proj_ was greater than that of *L*/*D* = 26.

The velocity of the fractured rods in the z-axis direction has a certain degree of reduction. The velocity reduction rate of fractured rods in the z-axis direction can be quantified by −∆*V_z_*/*V_proj_* value. With the increase of the long diameter ratio (*L*/*D*), −∆*V*_z_/*V*_proj_ increased, suggesting that the velocity of the fractured rods in the z-axis direction decreases. Primarily owing to the increase of *L*/*D*, the number of fractured rods *n* increased. With decreasing *L*_max_/*L*, the mass of a single fractured rod decreased, and the velocity of the fractured rods increased after interacting with LEFP.

The −∆*P*_z_/*P*_proj_ value reflects the momentum change rate of the fractured rods in the z-axis direction. The momentum reduction rate of the fractured rods in the z-axis direction decreased first and then increased with the increase of *L*/*D*. The value of −∆*P*_z_/*P*_proj_ at θ = 60° was greater than that at θ = 30°. Since the value of −∆*P*_z_/*P*_proj_ is related to the fractured rod mass $m_{i}$ and ∆*V*_z_, it can be seen that the change trend of the −∆*P*_z_/*P*_proj_ value is in line with the variation trends of the −∆*L*/*L* and −∆*V*_z_/*V*_proj_.

*H*_xz_ is determined by the rotation angle and movement velocity of the fractured rods around the center of mass. The fractured rod rotates clockwise around the center of mass when the angular velocity is positive, and vice versa. Δ*H*_xz_/*LP*_proj_ reflects the rotation amplitude of the fractured rod. It can be seen from Figure 13 that the rotation amplitude of the fractured rods at θ = 60° was higher than that at θ = 30°. The rotation amplitude of the fractured rods determines the size of the damaged area of the backplane. The broader the rotation amplitude, the larger the damaged area of the backplane.

### 4.3. The Velocity Effect of the Long Rod

**Figure 14:**
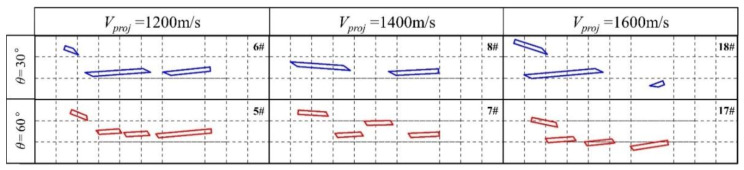
Location pattern of the residual fractured rod after the interaction of long rods having different velocities with different interception angles of LEFP (26 ms).

Figure 14 shows high-speed camera screenshots of a long rod with a *L*/*D* ratio of 35 at velocities of 1200, 1400, and 1600 m/s, respectively, at 26 ms after the signal board is triggered (5–8, 17, 18 round test results). It can be seen that with the increasing velocity of the long rod, the length of the middle-fractured rods increased. This result is primarily attributed to the increase in the velocity of the long rod and the increase in the interaction time difference of the adjacent LEFPs, resulting in the intersection backward of the LEFP with the long rod. As the length of the middle fractured rods increased, the angular momentum of the center fractured rods decreased, and the penetration depth increased.

**Figure 15:**
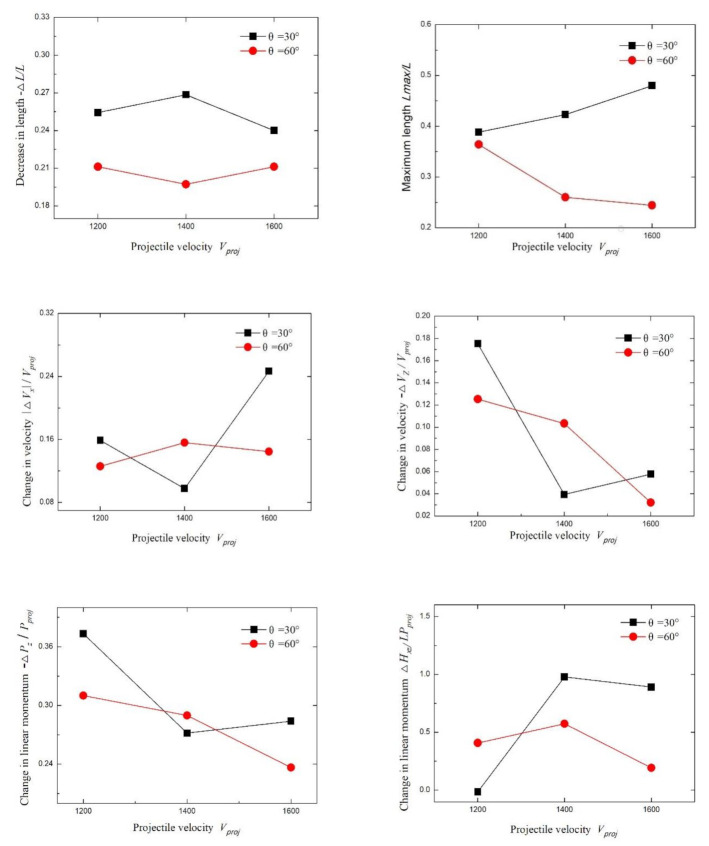
Influence of the projectile velocity *V*_proj_ at different inclination angles.

Figure 15 shows the fractured rods performance curve at different long rod velocities. It can be seen that the increase in the velocity of the long rod affected −∆*L*/*L* slightly. When θ = 30°, −∆*L*/*L* was higher than that of θ = 60°. In other words, when θ was small, the length loss rate of the fractured rod −Δ*L*/*L* was significant.

The value of *L*_max_/*L* at θ = 30° was higher than that at θ = 60°. When θ = 30°, the *L*_max_/*L* value increased as the velocity of the rod increased. When θ = 60°, the *L*_max_/*L* value decreased with the increasing velocity of the rod. When θ = 30°, the velocity of the long rod increased, whereas the time interval between adjacent LEFPs intercepting long rods remained constant, resulting in an increase in the length of the middle fractured rod and an increase in the *L*_max_/*L* value. When θ = 60°, the time interval was smaller than that at θ = 30°. In the meantime, the velocity of the long rod increased, resulting in a more uniform length of the fractured rods and decreased value of *L*_max_/*L*.

The |Δ*V*_x_|/*V*_proj_ value increased with the increase of the long rod velocity, indicating that the higher the velocity of the long rod, the less the stability of the fractured rods after being impacted by LEFPs. When θ = 30° and *V*_proj_ = 1400 m/s, the |Δ*V*_x_|/*V*_proj_ value exhibited some special characteristics. As can be seen from the images in Figure 15, the head fractured rod in the front section was also impacted by LEFP. In the meantime, the abrasion on tip appeared. This resulted in a small Δ*V*_x_ value of the fractured rod in the front section, as well as a small |Δ*V*_x_|/*V*_proj_ value. When θ = 60°, the |Δ*V*_x_|/*V*_proj_ value was less than that at θ = 30°, suggesting that the smaller the interception angle of LEFP, the more favorable it is for the fractured rods to rotate.

The value of −∆*V*_z_/*V*_proj_ decreased with the increase of the velocity of the long rod, which is primarily due to the fact that the fractured rod maintains inertial motion after the interaction with LEFP, which decreased the reduced value of the fractured rods velocity in the z-axis direction.

The value of −∆*P*_z_/*P*_proj_ showed a downward trend with the increase of the rod velocity. The changing trend was the same as that of −∆*V*_z_/*V*_proj_.

The value of Δ*H*_xz_/*LP*_proj_ was the maximum when *V*_proj_ = 1400 m/s regardless at θ = 30° or 60°, but diminished when the long rod velocity was 1200 or 1600 m/s. It can be seen that when LEFP intercepted a long rod at a certain velocity, the value of the angular momentum change of the fractured rods in the *XZ* plane was the maximum at the long rod velocity of about 1400 m/s, indicating that the rotation angle of the fractured rods and the spread range were large. It also can be seen that the value of Δ*H*_xz_/*LP*_proj_ was less impacted by the velocity of the rod at θ = 60° but more influenced by the velocity of the rod at θ = 30°.

### 4.4. Effect of LSC Spacing

**Figure 16:**
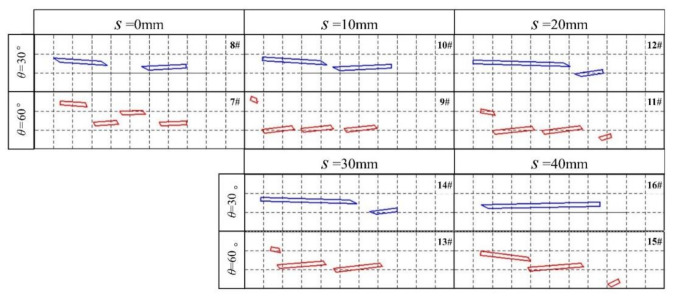
Location pattern of the residual fractured rods after the interaction of long rods with different interception angles of LEFP and LSC spacing (26 ms).

Figure 16 shows images of the fractured rods, where after 26 ms the long rod hit the signal board and interacted with LEFP with different spacing. The velocity of the long rod was 1400 m/s, the length-to-diameter ratio was 35, and the spacing of the linear shape charge was 0, 10, 20, 30, and 40 mm. With the increasing LSC spacing, the mutual interference in the detonation process is reduced, which is more favorable for the stable formation of LEFP. On the other hand, with the increasing LSC spacing, the length of the fractured rod changed from the isometric length to significantly different lengths with the reduced fractured rod amount. The main reason for this phenomenon is that after increasing the LSC spacing, the time interval between adjacent LEFP intercepting long rods increases, which reduces the number of fractured rods.

**Figure 17:**
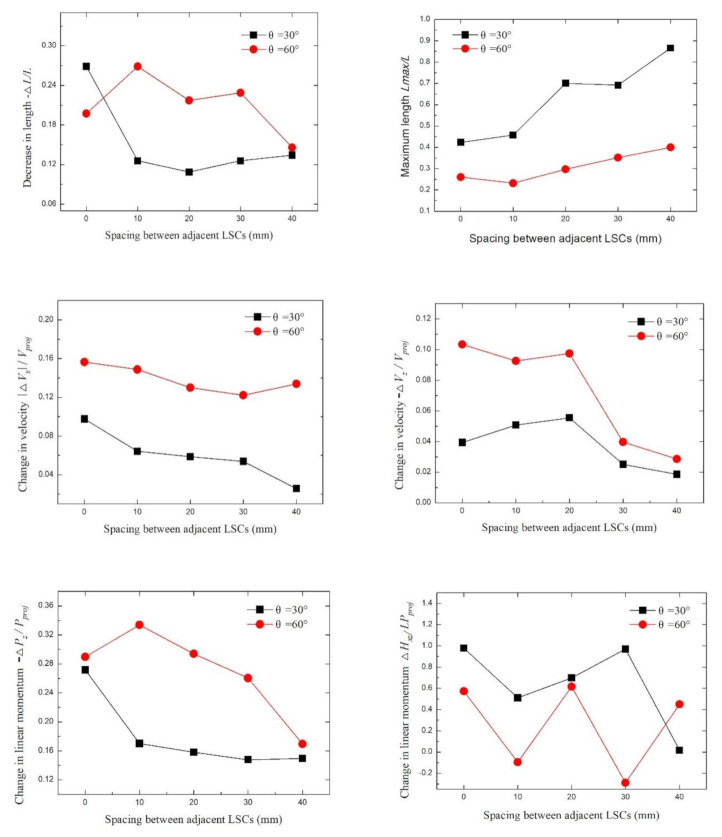
Influence of the LSC spacing at different inclination angles.

Figure 17 shows the curve of fractured rods after the interaction of LSCs having different spacings with the long rod. The value of −Δ*L*/*L* basically decreased with the increase of LSC spacing. When θ = 60°, there were more fractured rods than when θ = 30°, so the value of −∆*L*/*L* was higher. The −∆*L*/*L* value depends not only on the number of fractured rods, but also on whether the head or tail of the long rod is abraded by LEFP. In the case of *s* = 0, in the eighth test, when LEFP hit the long bar at an interaction angle of 30°, the head of the long rod fractured and abraded, resulting in a high value of −∆*L*/*L*. Similarly, in the case of *s* = 10, when LEFP hit the long pole at an inclination angle of 60°, one of the LEFPs and the tail of the long rod were abraded, so the −∆*L*/*L* value was also higher than when *s* = 0 and 20.

The *L*_max_/*L* value increased with the increase of the LSC spacing, indicating that the maximum length of the fractured rod increased. The maximum fractured rod length at θ = 30° was greater than the maximum fractured rod length at θ = 60°.

The |Δ*V*_x_|/*V*_proj_ value showed a downward trend with the increase of LSC spacing, which is attributed to various factors, including the reduced number of fractured rods, increased length, increased inertia, decreased longitudinal displacement, etc. The |Δ*V*_x_|/*V*_proj_ value at θ = 60° was significantly higher than that at θ = 30°.

The value of −Δ*V*_z_/*V*_proj_ decreased with the increase of the LSC spacing, indicating that the reduced value of the fractured rods on the z-axis velocity tended to be small, which is mainly due to the rise in the length of the fractured rod, the inertial motion of fractured rod, and the reduced effect of LEFP on the z-axis velocity of the fractured rods. The influence of LEFP on the velocity of the fractured rods at θ = 60° was greater than that at θ = 30°.

The value of −Δ*P*_z_/*P*_proj_ basically decreased with the increasing LSC spacing. This value is related to *m*_i_ and ∆*V*_i_. The value of −Δ*P*_z_/*P*_proj_ at θ = 60° was higher than that at θ = 30°.

The value of Δ*H*_xz_/*LP*_proj_ varied with the increase of LSC spacing, which exhibited a changing trend of a broken line. The value Δ*H*_xz_/*LP*_proj_ at θ = 30° was higher than that at θ = 60°. Δ*H*_xz_ depends on the rotation angle and movement velocity of the fractured rods around the center of mass. When the fractured rod rotates clockwise around the center of mass, the angular velocity is positive, and vice versa. Δ*H*_xz_/*LP*_proj_ reflects the fractured rods rotation amplitude. Only at θ = 60°, Δ*H*_xz_/*LP*_proj_ was a negative value when the spacing distance was 10 or 30 mm. In other words, the amplitude of the fractured rod rotating counterclockwise around the center of mass was greater than that of clockwise rotation.

## 5. Conclusions

This paper studies the deformation and fracture failure of a high-speed long rod intercepted by the LEFPs sequence. The following conclusions can be made:

- LEFP with a stable flight velocity of 850 m/s was formed after the LSC explosion. The LEFPs sequence could cut the high-speed long rod (speed about 1200~1600 m/s) in several segments (number about 2~4) and yaw the fractured rods.
- In this paper, the deformation and fracture failure of a high-speed long rod was quantified: The length loss rate of the fractured rods is as high as 27%, and the length of the longest fractured rod could be as low as 23% of the original length. The fractured rods rotated around the center of mass and the vertical speed change could reach up to 18% of the muzzle velocity of the long rod, and the greatest reduction of the horizontal speed and momentum could reach 37%.
- The increase in the interception angle and the reduction in the distance between the LEFPs sequence can increase the interaction time. The longer the interaction time, the more beneficial the failure of the long rod.

In this paper, the application of the LEFPs sequence solved the difficult problem of disabling the high-speed long rod. The quantitative analysis to the fracture failure of long rod had an important sense for studying the terminal penetration effect of the fractured rods.

## Figures and Tables

**Table 1:** Parameters of the LSC structure.

*R*_Liner_	δ_Liner_	*W*_Liner_	*R*_sh_	δ_sh_	*h*_ex_	*L*_Charge_
20 mm	3 mm	34 mm	43 mm	3 mm	12.5 mm	100 mm

**Table 2:** Results of the dynamic impact test.

Case	*L*/*D*	*h*	*V*_proj_	θ	*s*	*n*
1	20	150	1189	60°	0	3
2	20	175	1205	30°	0	2
3	26	188	1212	60°	0	3
4	26	165	1206	30°	0	2
5	35	169	1194	60°	0	4
6	35	174	1202	30°	0	2
7	35	176	1411	60°	0	4
8	35	149	1408	30°	0	2
9	35	179	1406	60°	10	4
10	35	166	1412	30°	10	2
11	35	187	1406	60°	20	4
12	35	160	1387	30°	20	2
13	35	210	1397	60°	30	3
14	35	170	1404	30°	30	2
15	35	185	1410	60°	40	3
16	35	150	1407	30°	40	1
17	35	194	1587	60°	0	4
18	35	145	1610	30°	0	2

**Table 3:** Change in length, velocity, linear momentum, and angular momentum.

Case	−Δ*L*/*L*	*L*_max_/*L*	Δ*V*_x_/V_proj_	Δ*V*_z_/*V*_proj_	Δ*P*_z_/*P*_proj_	Δ*H*_xz_/(*LP*_proj_)
1	0.245	0.41	0.11	−0.07	−0.30	0.45
2	0.22	0.67	0.19	−0.11	−0.31	−0.74
3	0.17	0.32	0.10	−0.07	−0.22	0.20
4	0.13	0.57	0.08	−0.15	−0.27	0.16
5	0.21	0.36	0.13	−0.13	−0.31	0.41
6	0.25	0.39	0.16	−0.18	−0.37	−0.02
7	0.20	0.26	0.156	−0.10	−0.29	0.57
8	0.27	0.42	0.10	−0.04	−0.27	0.98
9	0.27	0.23	0.15	−0.09	−0.33	−0.10
10	0.13	0.46	0.06	−0.05	−0.17	0.51
11	0.22	0.30	0.13	−0.10	−0.29	0.62
12	0.11	0.70	0.06	−0.06	−0.16	0.70
13	0.23	0.35	0.12	−0.04	−0.26	−0.29
14	0.13	0.69	0.05	−0.03	−0.15	0.97
15	0.15	0.40	0.13	−0.03	−0.17	0.45
16	0.13	0.87	0.03	−0.02	−0.15	0.02
17	0.21	0.24	0.14	−0.03	−0.24	0.19
18	0.24	0.48	0.25	−0.06	−0.28	0.89
